# In vivo length change of ligaments of normal knees during dynamic high flexion

**DOI:** 10.1186/s12891-020-03560-3

**Published:** 2020-08-15

**Authors:** Kenichi Kono, Shoji Konda, Takaharu Yamazaki, Sakae Tanaka, Kazuomi Sugamoto, Tetsuya Tomita

**Affiliations:** 1 Department of Orthopaedic Surgery, Faculty of Medicine, The University of Tokyo, 7-3-1 Hongo, Bunkyo-ku, Tokyo, 113-0033 Japan; 2 Department of Orthopaedic Biomaterial Science, Osaka University Graduate School of Medicine, 2-2 Yamada-oka, Suita, Osaka, 565-0871 Japan; 3 Deapartment of Information Systems, Faculty of Engineering, Saitama Institute of Technology, 1690 Fusaiji, Fukaya, Saitama, 369-0293 Japan

**Keywords:** Length change of ligaments, Kinematics, Squatting, Kneeling, Cross-leg

## Abstract

**Background:**

Few studies compared the length change of ligaments of normal knees during dynamic activities of daily living. The aim of this study was to investigate in vivo length change of ligaments of the normal knees during high flexion.

**Methods:**

Eight normal knees were investigated. Each volunteer performed squatting, kneeling, and cross-leg motions. Each sequential motion was performed under fluoroscopic surveillance in the sagittal plane. The femoral, tibial, and fibular attachment areas of the anterior cruciate ligament (ACL), posterior cruciate ligament (PCL), deep medial collateral ligament (dMCL), superficial medial collateral ligament (sMCL), and lateral collateral ligament (LCL) were determined according to osseous landmarks. After 2D/3D registration, the direct distance from the femoral attachment to the tibial or fibular attachment was measured as the ligament length.

**Results:**

From 20° to 90° with flexion, the ACL was significantly shorter during cross-leg motion than during squatting. For the PCL, dMCL, sMCL, and LCL, there were no significant differences among the 3 motions.

**Conclusion:**

The ACL was shorter during cross-leg motion than during squatting in mid-flexion. This suggests that the ACL is looser during cross-leg motion than during squatting. On the other hand, the length change of the PCL, MCL, and LCL did not change even though the high flexion motions were different.

## Background

Soft tissue balance such as ligament balance is one of the most important factors for successful total knee arthroplasty (TKA). In addition, ligament-retained TKA has increased worldwide. Therefore, surgeons now take more interest in the ligament balance of the knee. Many previous studies reported length change to the ligaments of normal knees [1–10]. However, some studies examined the in vitro condition [1, 5, 7]. Regarding in vivo studies, they examined static motions or simple active motions [2–4, 6, 8, 10, 11] such as leg flexion and extension. Several studies evaluated the length change of ligaments of normal knees during dynamic activities of daily living [9, 12–14]. Kernkamp et al. measured the in vivo anterolateral ligament length change in healthy knees during step-up and sit-to-stand motions under dynamic conditions [13]. Taylor et al. used a combination of magnetic resonance imaging (MRI), Motion Capture, and dual fluoroscopy to characterize relative strain in an anterior cruciate ligament (ACL) during both stance and swing phases of normal level walking [12]. Liu et al. measured the in-vivo length patterns of the deep medial collateral ligament (dMCL) and superficial medial collateral ligament (sMCL) during walking using dual fluoroscopy [9]. Rao et al. investigated the length changes of ACL and posterior cruciate ligament (PCL) during a single-legged lunge [14]. However, few studies directly compared the length change of ligaments of normal knees during dynamic high flexion activities of daily living.

Especially in Asia and the middle-east, people commonly bend their knees deeply in daily living, like sitting on the floor and praying. In Western countries, many people perform a squatting position while they play sports. In addition, several studies have demonstrated that the in vivo kinematics of normal knees during high-flexion activities are significantly different [15–17]. Therefore, it is important to investigate the length change during high-flexion motions all over the world. Furthermore, several reports have demonstrated that patients’ satisfaction with TKA has been greater with a medial pivot pattern, such as in normal knees [18, 19]. Therefore, we thought that it was necessary to evaluate the length change of ligaments of normal knees during high flexion to further increase patients’ satisfaction after ligament-retained TKA.

The aim of this study was to investigate in vivo length change of ligaments of normal knees during dynamic high-flexion activities. The hypothesis of this study was that the length change of ligaments of normal knees was different depending on the high flexion activities of daily living.

## Methods

A total of 8 normal knees (4 Japanese healthy male volunteers) were investigated. At the time of the investigation, their mean age was 41.8 ± 6.5 years, mean height was 170.3 ± 5.9 cm, and mean weight was 68.5 ± 9.7 kg. This study was approved by the ethics committee, and all of the volunteers provided written, informed consent prior to participation.

**Fig. 1:**
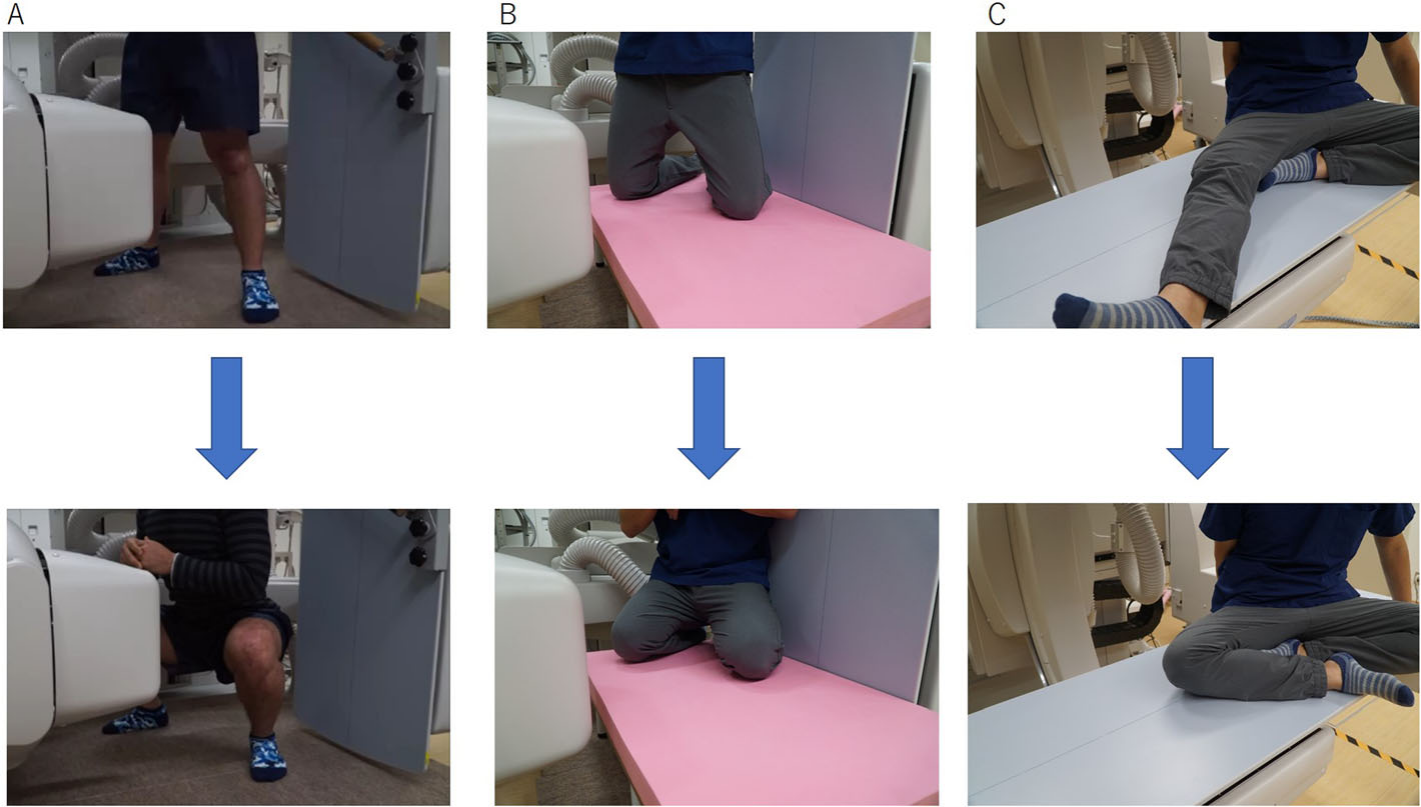
The fluoroscopic analysis. **a**: Squatting. **b**: Kneeling. **c**: Sitting cross-legged

Each volunteer was asked to perform squatting, kneeling, and sitting cross-legged (cross-leg) motions. The activities were performed under fluoroscopic surveillance in the sagittal plane (Fig. 1). The kneeling motion was measured from 100° to 150° with flexion. Sequential knee flexion was recorded as digital X-ray images (1024 × 1024 × 12 bits/pixel, 7.5-Hz serial spot images as a DICOM file) using a 17-in. flat panel detector system (C-vision Safire L; Shimadzu, Kyoto, Japan). On this system, acquired images were nondistorted and clear compared with the Image Intensifier system (Shimadzu). In addition, all images were processed by dynamic range compression, enabling edge-enhanced images. To estimate spatial position and orientation of the knee, a 2-dimensional/3-dimensional (2D/3D) registration technique was used [20, 21]. This technique is based on a contour-based registration algorithm using single-view fluoroscopic images and 3D computer-aided design (CAD) models. Three-dimensional bone models were created from computed tomography (CT) and used as CAD models for subsequent 2D/3D registration. The estimation accuracy of relative motion between 3D bone models was ≤1° in rotation and ≤ 1 mm in translation [22]. A local coordinate system at the bone model was produced according to previous studies [22].

We defined the femoral, tibial and fibular attachment areas of the ACL, PCL, dMCL, sMCL and lateral collateral ligament (LCL) according to the osseous landmark [8, 10, 23–31]. The accuracy of the attachment area is 0.7 ± 0.1 mm [31]. The collateral ligaments were each evenly divided into 3 portions (anterior (a), middle (m), and posterior (p)) [8, 10]. The direct distance from the femoral attachment to the tibial or fibular attachment was measured as the ligament length (L) using software (MATLAB, MathWorks, Natick, MA, USA). The ligament length at 0° of knee flexion during squatting was defined as the reference ligament length (L ref). The length change (ℯ) was calculated using an engineering strain formula (i.e. ℯ = (L – L ref) / L ref. × 100). Extension relative to the reference ligament length was denoted as positive, and shortening was denoted as negative (Video 1).

All data are expressed as means ± standard deviations (SD). Two-way analysis of variance (ANOVA) and post hoc pair-wise comparison (Bonferroni correction) were used to analyze differences in the length change of each ligament among squatting, kneeling, and cross-leg motions. *p*-value < .05 were considered significant.


**Additional file 1.**


## Results

### ACL

During squatting, the ACL shortened gradually up to an average of − 30.15% ± 4.8% from 0° to 120° with knee flexion. From 120° to 150° with flexion, it extended an average of up to − 12.85% ± 7.9%. During kneeling, the ACL did not change significantly from 100° to 130° with flexion. From 130° to 150° with flexion, it extended from − 38.8% ± 4.2% to − 31.9% ± 6.3%. During cross-leg motion, the ACL shortened gradually on average from 3.7% ± 6.2% to − 36.9% ± 4.3% from 0° to 110° with flexion. From 110° to 150° with flexion, it extended an average of up to − 24.9% ± 6.3%.

**Fig. 2:**
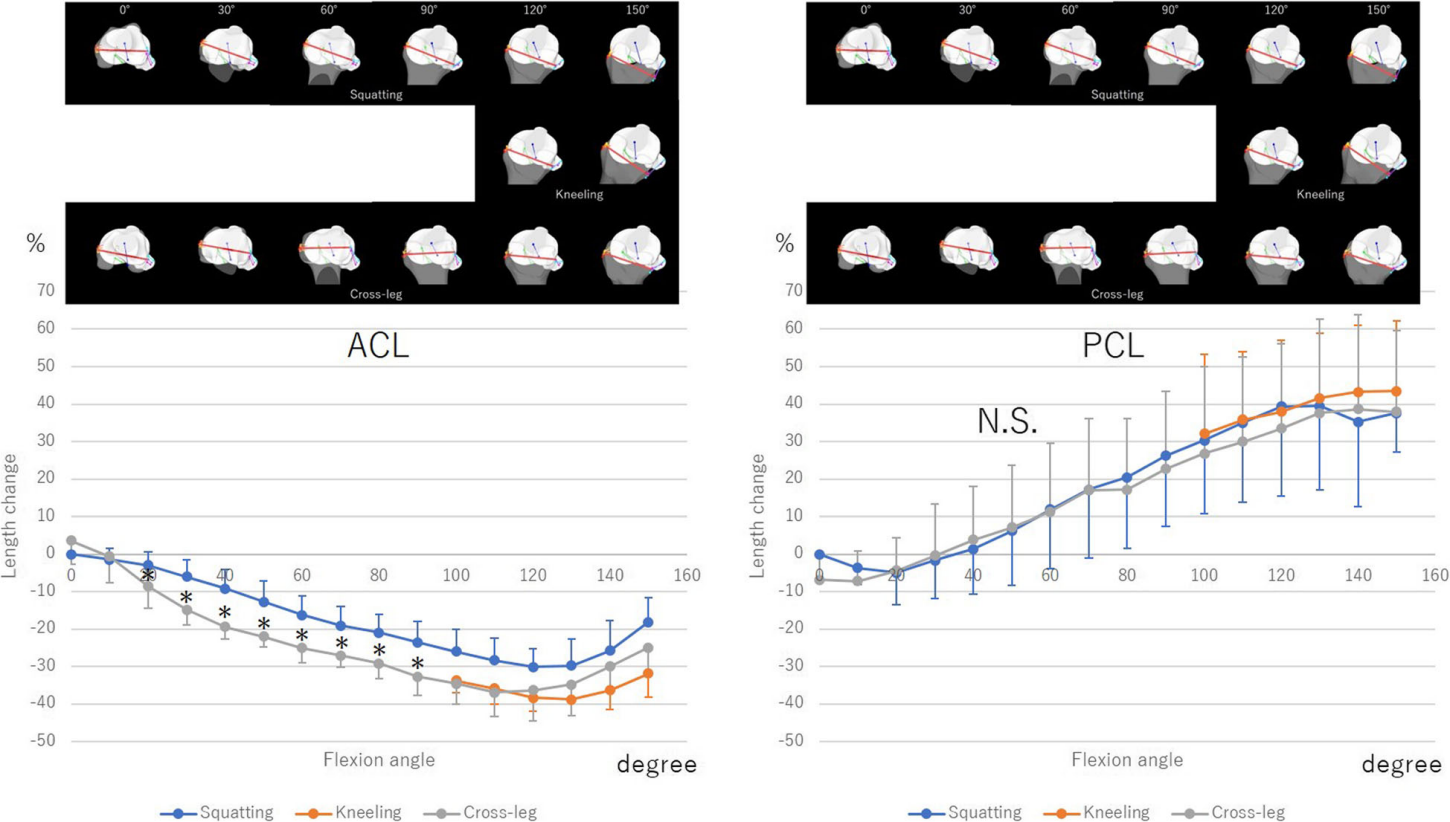
The length change of anterior cruciate ligament (ACL) and posterior cruciate ligament (PCL) during squatting, kneeling and cross-leg. Red line of the upper table indicates the surgical epicondyle line. *: Significant differences between squatting and cross-leg (*p* < 0.05)

The length change of the ACL during squatting was smaller than that during cross-leg motion from 20° to 90° with flexion (Fig. 2).

### PCL

The PCL extended gradually with flexion up to 37.6% ± 10.3% during squatting, 43.5% ± 18.8% during kneeling, and 37.9% ± 21.6% during cross-leg motion. Among the 3 deep bending activities, there was no significant difference (Fig. 2).

### MCL

Regarding dMCL, adMCL, and mdMCL extended from 0° to 120° (adMCL 42.6% ± 21.4, 45.5% ± 20.2, and 39.5% ± 18.8% during squatting, kneeling, and cross-leg motion, respectively; mdMCL 28.9% ± 21.0, 30.0% ± 19.0, and 21.5% ± 18.1% during squatting, kneeling, and cross-leg motion, respectively). From 120° to 150°, adMCL shortened 3.7% ± 19.3, 3.1% ± 4.7, and 16.6% ± 12.1%, during squatting, kneeling, and cross-leg motion, respectively, and mdMCL shortened 5.95 ± 18.1, 2.0% ± 4.6, and 15.2% ± 11.4% during squatting, kneeling, and cross-leg motion, respectively. Regarding sMCL, asMCL extended 10.1% ± 7.2% during squatting, 9.5% ± 7.1% during kneeling, and 9.2% ± 7.9% during cross-leg motion from 0° to 100° with flexion. From 100° to 150°, they shortened 5.6% ± 8.1, 5.1% ± 2.6 and 11.3% ± 5.4% during squatting, kneeling, and cross-leg motion, respectively. There were no significant differences in msMCL, psMCL, and pdMCL.

**Fig. 3:**
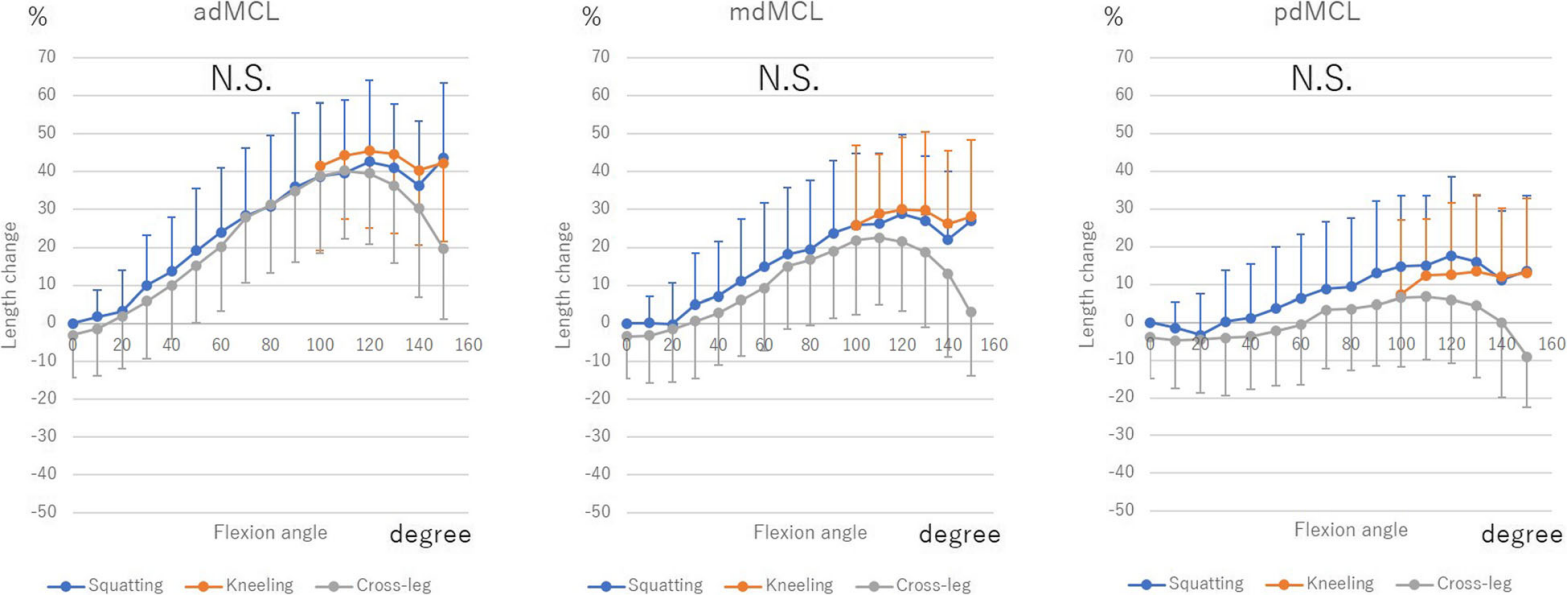
The length change of deep medial collateral ligament (dMCL) during squatting, kneeling and cross-leg. The length change was calculated as the length change rate from the ligament length at 0° of knee flexion during squatting. N.S.: Not significant

**Fig. 4:**
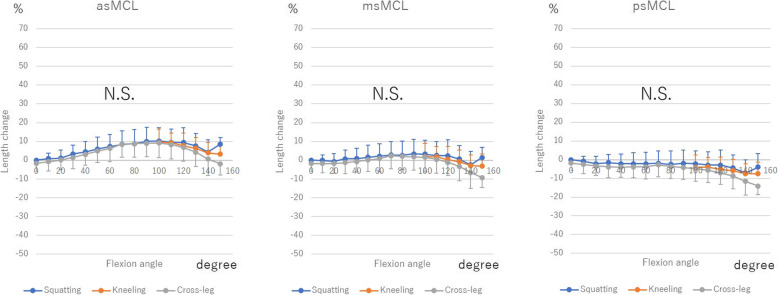
The length change of superficial medial collateral ligament (sMCL) during squatting, kneeling and cross-leg. The length change was calculated as the length change rate from the ligament length at 0° of knee flexion during squatting. N.S.: Not significant

Among the 3 deep bending activities, there were no significant differences in both dMCL and sMCL (Figs. 3 and 4).

### LCL

aLCL extended with flexion (up to 13.7% ± 25.2% during squatting, 12.7% ± 16.7% during kneeling, and 23.7% ± 22.7% during cross-leg motion). On the other hand, there were no significant differences in mLCL and pLCL.

**Fig. 5:**
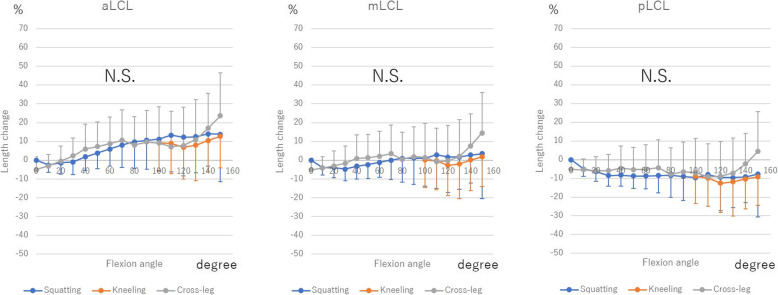
The length change of lateral collateral ligament (LCL) during squatting, kneeling and cross-leg. The length change was calculated as the length change rate from the ligament length at 0° of knee flexion during squatting. N.S.: Not significant

Among the 3 deep bending activities, there were no significant differences (Fig. 5).

## Discussion

A most important finding in this study was that the ACL was shorter during cross-leg motion than during squatting in mid-flexion. This fact suggested that the ACL is looser during cross-leg motion than during squatting. In other words, the ACL might be easy to affect by differences in flexion motions. Hence, ACL-preserved TKA might be able to reproduce the ACL length difference between squatting and cross-legged sitting. On the other hand, regarding length changes of the PCL, MCL, and LCL, there were no significant differences among the 3 motions. This suggested that the length changes of PCL, MCL, and LCL do not change even though the high-flexion motions are different. In addition, the ACL might be the most easily affected ligament of the knee in mid-flexion. The previous study demonstrated that the effect of coronal plane knee motion on cruciate ligament was larger than that of collateral ligament [32]. Additionally, ACL during Closed-Kinetic-Chain activities largely elongated, while that during Open-Kinetic-Chain activities slightly elongated [33]. Therefore, the ACLs have changed in cross-legged motion. In high flexion, the length change of the ACL was also not significantly different among the 3 motions. This suggested that any length changes of the ligaments do not change even though the flexion motions are different in high flexion. Therefore, regarding the ligament balance in bi-cruciate retaining TKA, the difference between the three activities might be low in high flexion.

From early flexion to mid-flexion, the ACL shortened with flexion. On the other hand, it extended slightly during high flexion. These tendencies were similar among the 3 motions. Additionally, the length change pattern with flexion was similar to that seen in previous in vivo studies using transducers [2, 3]. However, the length change of previous studies was less than 10%, while the length changes in the present study were more than 30%. Many factors such as ethnicity, gender, age, body mass index, or method of analysis contribute to the difference. Especially the method of analysis, the previous studies were analyzed from 0° to 90° of flexion. While this study was evaluated from 0° to 150° of flexion. Moreover, another previous study that evaluated the ligament length change using similar methods reported the ACL elongated more than 30% from 120° to 0° of flexion [14]. Hence, it was thought that the data in the current study was appropriate.

The PCL extended with flexion in the present study. Previous studies that investigated in vivo length change of the PCL using static methods also indicated the same pattern, including the absolute values [4, 34, 35]. This fact suggested that the PCL is commonly affected in high flexion, and the length change during dynamic motion was similar to that during static motion.

Regarding the MCL, adMCL, mdMCL, and asMCL extended from early flexion to mid-flexion. This trend was similar to previous studies [8, 10]. These facts suggested that the anterior portion of the MCL is easily affected in mid-flexion. Furthermore, the deep layer of the MCL might be easily affected in mid-flexion, because two thirds of the portion extended. During TKA for varus knee, we usually release the MCL to modify the soft tissue balance. Releasing the anterior and deeper layers of the MCL might be effective to modify mid-flexion balance.

Regarding the LCL, the anterior portion extended with flexion. This trend was similar to the previous study [10]. These facts suggested that the anterior portion could be affected in the lateral soft tissue balance, especially in high flexion. The high SD might mean high variation. Therefore, it might be impossible to detect the difference statistically because of the high variation. While this suggested that there is a high variation of LCL length change regardless of the type of high flexion activities in normal knees.

The high SDs in this study suggested many inter-subject variations in normal knees. However, the trends were similar to previous studies [8, 10, 36]. Therefore, it is thought that the results of this study were appropriate.

Several limitations of this study need to be discussed. First, the present study only analyzed Japanese male subjects. Female subjects or other races might display different length changes. Second, the number of volunteers involved was small. Therefore, the present results might not be generalizable to the general population. Third, only normal knees were evaluated. The length changes to ligaments of osteoarthritis knees and knees after TKA might be different from those of normal knees. Therefore, we will investigate the length changes to ligaments of their knees in our next study. Forth, single bundles of ACL and PCL were analyzed in the current study. Double bundles analysis might show different results. Fifth, the length of the direct line between the attachment areas was defined as the ligament length in this study. While, some studies used shortest three-dimensional wrapping path because the MCL wraps along the surfaces of the tibia and femur [8, 10]. Therefore, the ligament length of MCL might be shorter than that of the previous studies [8, 10]. However, a previous computer simulation study that used the straight line for ligament reported the ligament force was similar to the real ligament [37]. In addition, the ligament length change during squatting was similar to the previous studies [8, 10]. Therefore, it is thought that the length change of MCL in this study is appropriate. Sixth, although each volunteer practiced several times before the fluoroscopic analysis, the kinematics was measured only one time per activity due to the radiation dose limit. An intra-subject variation may also affect the results.

## Conclusions

The ACL was shorter during cross-leg motion than during squatting in mid-flexion. This suggests that the ACL is looser during cross-leg motion than during squatting. On the other hand, the length changes of the PCL, MCL, and LCL did not change even though the high-flexion motions were different.

## Data Availability

The datasets analysed during the current study are available from the corresponding author on reasonable request.

## Abbreviations

TKA: Total knee Arthroplasty
ACL: Anterior cruciate ligament
PCL: Posterior cruciate ligament
dMCL: Deep medial collateral ligament
sMCL: Superficial medial collateral ligament
2D/3D: 2-dimensional/3-dimensional
CAD: Computer-aided design
CT: Computed tomography
LCL: Lateral collateral ligament
MRI: Magnetic resonance imaging
a: Anterior
m: Middle
p: Posterior
L: Ligament length
L ref.: Reference ligament length
SD: Standard deviations
ANOVA: Analysis of variance
